# Supplementary material for: Immune signaling of Litopenaeus vannamei c-type lysozyme and its role during microsporidian Enterocytozoon hepatopenaei (EHP) infection
Source: PLoS Pathog. 2024 Apr 29;20(4):e1012199. doi: 10.1371/journal.ppat.1012199 (PMC11081493; doi:10.1371/journal.ppat.1012199)
Supplement: S1 Fig — The rLvLyz-c was analyzed on 15% SDS-PAGE and the western blot was performed using an anti-his antibody. rLvLyz-c was expressed from the E. coli stain Rosetta(DE3)pLysS transformant with (+) and without (−) IPTG induction. Lanes (P) are purified proteins. Lanes M are standard protein size markers. (DOCX) [file ppat.1012199.s001.docx]

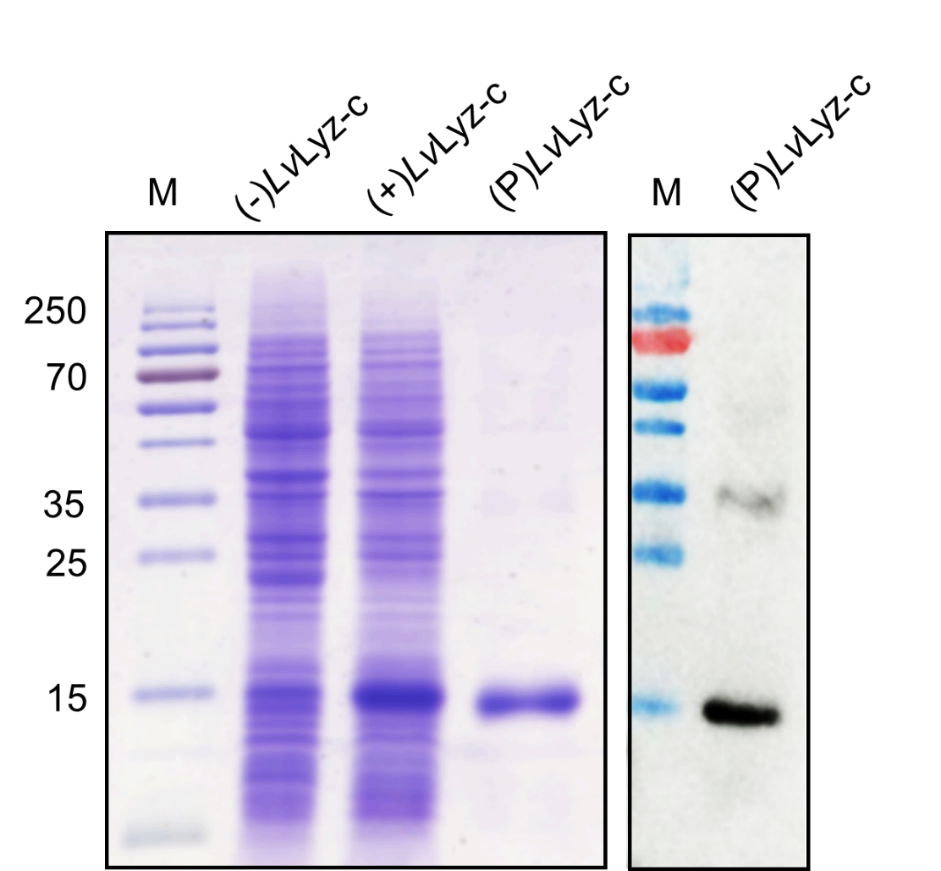


**Fig S1. The expression and purification of recombinant *Lv*Lyz-c.** The r*Lv*Lyz-c was analyzed on 15% SDS-PAGE and the western blot was performed using an anti-his antibody. r*Lv*Lyz-c was expressed from the *E. coli* stain Rosetta(DE3)pLysS transformant with (+) and without (−) IPTG induction. Lanes (P) are purified proteins. Lanes M are standard protein size markers.
