# Supplementary material for: Immune signaling of Litopenaeus vannamei c-type lysozyme and its role during microsporidian Enterocytozoon hepatopenaei (EHP) infection
Source: PLoS Pathog. 2024 Apr 29;20(4):e1012199. doi: 10.1371/journal.ppat.1012199 (PMC11081493; doi:10.1371/journal.ppat.1012199)
Supplement: S1 Table — (DOCX) [file ppat.1012199.s002.docx]

**Table S1.** Primers used in this study

| Primer name | Sequence (5′→3′) | Purpose |
| --- | --- | --- |
| *EF-1α*-F | CTTGATTGCCACACTGCTCAC | qRT-PCR |
| *EF-1α*-R | TCTCCACGCACATAGGCTTG | qRT-PCR |
| *EhSSU rRNA*-F | ATTAGACACCGCTGTAGTTC | qRT-PCR |
| *EhSSU rRNA*-R | GTTATTGCCTTCTCCCTC | qRT-PCR |
| *LvTLR2*-F | TCCTTGATATCCGTGGGAAC | qRT-PCR |
| *LvTLR2*-R | CCTCCGTCACGTCTATCAG | qRT-PCR |
| *LvMyD88*-F | GTGCACCAGAGTCATTGTAG | qRT-PCR |
| *LvMyD88*-R | GGGAGTGGCAGAAACTTATC | qRT-PCR |
| *LvDorsal*-F | TCACTGTTGACCCACCTTAC | qRT-PCR |
| *LvDorsal*-R | GGAAAGGGTCCACTCTAATC | qRT-PCR |
| *LvDome*-F | TCAGACAGGAGGTCTCATAC | qRT-PCR |
| *LvDome*-R | GTACCAGTGTGAAGCCTTAC | qRT-PCR |
| *LvJAK*-F | TACCCTGGTCTACGCTATAC | qRT-PCR |
| *LvJAK*-R | TGAGACGGTAGTACCCATTC | qRT-PCR |
| *LvSTAT*-F | CCAGTGCTTGAACACTGAAC | qRT-PCR |
| *LvSTAT*-R | GGCTTTGAATGTGGGATAGG | qRT-PCR |
| *LvLyz-c*-F | CCCATGTTCCGATCTGATGTC | qRT-PCR |
| *LvLyz-c*-R | CACTTGCTGTTGTAAGCCACC | qRT-PCR |
| *LvALF1*-F | GTCCTCCGTGATGAGATTACTCTG | qRT-PCR |
| *LvALF1*-R | TTACTTCAATGGCAGGATGTGG | qRT-PCR |
| *LvPEN3*-F | CACCCTTCGTGAGACCTTTG | qRT-PCR |
| *LvPEN3*-R | AATATCCCTTTCCCACGTGAC | qRT-PCR |
| dsDOME-F | GTTCTTCCTACGAAGCCTACTGC | RNAi |
| dsDOME-R | ATGTATTGAACCCTGACTGGATTTG | RNAi |
| *dsRNA-DOME-T7-*F | GGATCCTAATACGACTCACTATAGGGTTCTTCCTACGAAGCCTACTGC | RNAi |
| *dsRNA-DOME-T7-*R | GGATCCTAATACGACTCACTATAGGATGTATTGAACCCTGACTGGATTTG | RNAi |
| *dsRNA-TLR2-*F | TGTGGATGCCAATGATCTTATC | RNAi |
| *dsRNA-TLR2-*R | GTAATCTGCTGCTGAATGTAGG | RNAi |
| *dsRNA-TLR2-T7-*F | GGATCCTAATACGACTCACTATAGGTGTGGATGCCAATGATCTTATC | RNAi |
| *dsRNA-TLR2-T7-*R | GGATCCTAATACGACTCACTATAGGGTAATCTGCTGCTGAATGTAGG | RNAi |
| *dsRNA-GFP*-F | ATGGTGAGCAAGGGCGAGGA | RNAi |
| *dsRNA-GFP*-R | TTACTTGTACAGCTCGTCCA | RNAi |
| *dsRNA-GFP*-T7-F | TAATACGACTCACTATAGGATGGTGAGCAAGGGCGAGGA | RNAi |
| *dsRNA-GFP*-T7-R | TAATACGACTCACTATAGGTTACTTGTACAGCTCGTCCA | RNAi |
| r*Lv*Lyz-c-NcoI-F | ATACCATGGGCCATCATCATCATCATCACTCCGACGCCAAGGTCTTC | Recombinant protein expression |
| r*Lv*Lyz-c-XhoI-R | CTCGAGCACCTAGAACGGGAAGACAGAG | Recombinant protein expression |
| HindIII_Pro_*LvLyz-c*_F | AAGCTTAACCGCACACTCATACAC | Luciferase reporter assay |
| NcoI_Pro_*LvLyz-c*_R | CCATGGCAACCTGAAATCATCTGCAATC | Luciferase reporter assay |
| HindIII_*LvSTAT*_F | AAGCTTATGTCGTTGTGGAACAGAGCAC | Luciferase reporter assay |
| BamHI_*LvSTAT*_R | GGATCCTGAAAAGTCTGAGAGGACATTTGC | Luciferase reporter assay |
