# Supplementary material for: Immune signaling of Litopenaeus vannamei c-type lysozyme and its role during microsporidian Enterocytozoon hepatopenaei (EHP) infection
Source: PLoS Pathog. 2024 Apr 29;20(4):e1012199. doi: 10.1371/journal.ppat.1012199 (PMC11081493; doi:10.1371/journal.ppat.1012199)
Supplement: Attachment — Submitted filename: Response to reviewers_PLoS_pathogens_LyzC_R1.docx [file ppat.1012199.s004.docx]

**Part I - Summary**
Please use this section to discuss strengths/weaknesses of study, novelty/significance, general execution and scholarship.

**Reviewer #1:** This paper seeks to provide insight into the role of shrimp lysozyme-c type (LvLyz-c) in the immune response to infection by the microsporidian pathogen Enterocytozoon hepatopenaei (EHP). Although LvLyz-c was upregulated upon EHP infection, the authors investigated the expression of immune signaling pathways that could regulate LvLyz-c expression during infection. They conclude that the shrimp Domeless promotes LvLyz-c expression and inhibits EHP proliferation. By performing luciferase assays, the authors conclude that LvLyz-c is regulated by the LvSTAT, supporting their claim that JAK/STAT signaling is required for LvLyz-c expression during EHP infection. By heterologous expression of LvLyz-c in bacteria, the authors determine that LvLyz-c has antibacterial and anti-fungal properties. In addition, using the recombinantly expressed LvLyz-c, the authors conclude that LvLyz-c destroys the chitin-containing endospore of EHP and reduces polar tube firing.
As EHP is a significant problem for commercial shrimp farms, it is commendable that the authors investigate the immune response of shrimp to EHP to identify host targets for infection intervention. However, the authors should address many major concerns (detailed below).

**Reviewer #2:** Sangklai et al. provide an intriguing study that suggests a chitinase activity of invertebrate Lysozyme from shrimp. In reviewing this article, I've come to learn that anti-Candida effects of lysozyme are well-described using vertebrate lysozyme, but less description of antifungal activity has been given for invertebrate lyszyome families (to my knowledge). This study therefore provides a valuable contribution to the literature on invertebrate lysozyme. The authors have also done a good job to present a concise and convincing series of experiments that nicely characterize the anti-Candida effects of LvLyz-c, including an investigation of precisely how it affects Candida endo- and exospore layers.

Overall, the study is convincing and sticks to presenting the core observations of the question at hand. I have only minor comments. A couple of my suggestions arise from my own work, so I'll forego anonymity here and note my identity as Mark A. Hanson.

We thank both reviewers for constructive and useful comments. We have addressed all the concerns raised by both reviewers and modified the manuscript accordingly. For more details, please find our responses below.

**Part II – Major Issues: Key Experiments Required for Acceptance**
Please use this section to detail the key new experiments or modifications of existing experiments that should be absolutely required to validate study conclusions.

Generally, there should be no more than 3 such required experiments or major modifications for a "Major Revision" recommendation. If more than 3 experiments are necessary to validate the study conclusions, then you are encouraged to recommend "Reject".

**Reviewer #1:**

1) With the cohabitation setup, having a standardized amount of EHP spores used across infections seems impossible. This is a major concern when interpreting any data generated from infected shrimp.

This is our limitation when working with EHP. It is widely accepted in the field of EHP study to use co-habitation assay as an infection model; for example,

[1] Thepmanee et. al, 2023 (DOI:[10.1038/s41598-023-47114-8](https://www.nature.com/articles/s41598-023-47114-8))

[2] Sukonthamarn et. al, 2023 (DOI:[10.1016/j.fsi.2023.108710](https://www.sciencedirect.com/science/article/abs/pii/S1050464823001961))

[3] Salachan et. al, 2016 (DOI:[10.1186/s12917-016-0923-1](https://pubmed.ncbi.nlm.nih.gov/28056950/))

In our laboratory, we have tried to add purified EHP spores into 200L aquariums so that we can control the amount of the parasites. However, the EHP infection rate is much less than using the co-habitation method. Therefore, the co-habitation assay is more feasible to use as a EHP infection model.

2) For all qRT-PCR results, the authors normalized the gene expression in infected shrimp to uninfected shrimp. Without seeing the raw Ct values for these experiments, it is difficult to conclude that the expression of a given gene changed due to the infection or just time. The authors should identify a gene that does not change expression due to infection or time and normalize to that for both infected and uninfected samples. It seems they did include EF1 as a control, so it is unclear why it was not used for normalization. In addition, the amount of cDNA used per reaction should be included in the materials and methods.

[1] We have already used the expression of the EF1ɑ gene for normalization in our analyses. 2^–∆∆Ct^ method was used to analyze the relative changes in gene expression. We thank reviewer 1 for pointing this out. We have now included how we analyzed the gene expression level into more details in Line 338-340.

[2] Regarding the amount of cDNA in the reaction, we used 10 ng of the cDNA as a template. We have now included this detail in Line 330-331 and Line 356.

[3] Reviewer 1 concerned that “*Without seeing the raw Ct values for these experiments, it is difficult to conclude that the expression of a given gene changed due to the infection or just time*”. We would like to confirm the change of the gene expression is due to the infection, not time. In the healthy shrimp, the ct values are consistent at all cohabitation time points.

3) It is unclear why the authors did not quantify EHP expression when RNAi against LvLyz-C is performed. The expression of multiple genes will decrease by RNAi against LvDome, including Lv-Lyz-c, and the authors cannot conclude that Lv-Lyz-c inhibits infection without knocking it down.

We thank reviewer 1 for this concern. Our initial goal is to identify which immune signaling pathways are responsible for *Lv*Lyz-c production. Hence, this prompted us to knock down *LvDome* and *LvTLR2* receptors. Even though the idea of knocking down *Lv*Lyz-c directly seems appropriate, this experiment could take months and it is beyond the scope of this study.

To further confirm that the *Lv*Lyz-c could inhibit the EHP infection, we have planned to overexpress the *Lv*Lyz-c in shrimp by supplementing the *Lv*Lyz-c into the feeds. Our preliminary data suggests that shrimp fed with *Lv*Lyz-c significantly reduce the EHP copy number compared to the normal feed group.

4) The luciferase assay of LvSTAT and Lv-Lyz-c promoter demonstrates a 2.88-fold increase in activity, which is relatively low. The authors should quantify luciferase activity when only the pGLE3 plasmid is present to eliminate the possibility that LvSTAT is not required for the slight increase in activity reported. In addition, it is unclear why the authors did not normalize to Renilla for each condition in the assay.

[1] We would like to confirm that we normalize our luciferase activity with Renilla. To prevent the confusion to the readers, we have now edited the Y-axis label of the Figure 3B to “Relative luciferase activity (Firefly/Renilla)”.

[2] Regarding the concern that slight increase of the luciferase activity may be due to the activity of the reporter. We have performed the experiment to include pGL3 plasmid with the *LvLyz-c* promoter. The result shows that the increase of luciferase indeed comes from the binding between *Lv*STAT and *LvLyz-c* promoter (as shown in the graph below). We have now included this control in a modified version of Figure 3B and main text Line 185.

[3] Regarding the comment that the 2.88-fold increase is relatively low, the binding of proteins or transcription factors to promoter regions can be varied. Some proteins can bind tighter than others. Soponpong et. al, 2022 (DOI:[10.3389/fimmu.2021.818267](https://doi.org/10.3389%2Ffimmu.2021.818267)) has shown that different transcription factors bind to the promoter with different affinities as assessed by the luciferase assay. For example, binding of transcription factor *Pm*IRF and the promoter of IFN-β increased the luciferase activity by 2.29 fold. Hence, our results could suggest that STAT weakly binds to the *LvLyz-c* promoter.

5) To quantify the endospore layer size, the authors draw a line between the exospore and endospore and quantify its length. A more accurate measurement would be to calculate the area of the endospore in treated vs untreated spores.

We thank reviewer 1 for this great suggestion. We have quantified the area of both endospore and exospore. The results are consistent with our previous measurement. We have now included this result in Figure 5E.

**Reviewer #2:** (No Response)

**Part III – Minor Issues: Editorial and Data Presentation Modifications**
Please use this section for editorial suggestions as well as relatively minor modifications of existing data that would enhance clarity.

**Reviewer #1:** (No Response)

**Reviewer #2:**
Minor comments:

Line 148: “This highlighted that LvLyz-c is one of the important AMPs to fight against the EHP infection.”

I would discourage this line of thinking. My own work has shown that AMPs are co-regulated due to a need for concise gene regulation, but gene expression should not be inferred as evidence of importance or even of relevance.
That’s not to say induced genes can’t be important, of course they can. The authors characterisation of LvLyz-c is also convincing. But there are tens to hundreds of induced genes after a given infection like EHP, and many of those genes likely contribute little, if anything, to host resistance/tolerance to EHP. I recently wrote a review describing this point in detail reflecting on studies by myself and others using Drosophila, nematodes, and more, available as a preprint and soon to be published in Philosophical Transactions of the Royal Society B. See: <https://ecoevorxiv.org/repository/view/6138/>
The authors could simply reword their sentence to avoid implying induction is evidence of effect. I really want to emphasize that I don't want to coerce a citation here. I just ask the authors to avoid this logical leap that induction is somehow evidence of function against the inducer.

We thank reviewer 2 for this concern. We have now modified Line 148 to tone down the claim and avoid this logical leap.

Fig. 1: it’s striking how the temporal patterns of LvALF1, LvLyz-c, LvDOME, LvSTAT etc… have such tight bounds to their variance, but 11 days post-cohabitation seems to be much lower than both 9 and 15 days post-cohabitation.
First a technical question: can I just confirm how the samples were collected? What is their level of study design independence?
Second: if the authors argue these are truly independent samplings, then could I ask the authors to explain how they imagine such up-and-down induction of immunity might be driven?

In Figure 1 and 2, three biological replicates or three individual shrimp were collected from the tank for each cohabitation timepoint. The up-and-down expression pattern observed here could reflect the cycles of the immune induction. This up-and-down pattern seems to be consistent with studies on other immune related genes in shrimp. For example,

[1] Janewanthanakul et. al., 2020 (DOI:[10.1016/j.dci.2019.103476](https://www.sciencedirect.com/science/article/pii/S0145305X19303295))

[2] Li et. al, 2018 (DOI:[10.1371/journal.ppat.1007109](https://pubmed.ncbi.nlm.nih.gov/30256850/))

Line 183: could the authors indicate where this STAT binding site motif comes from with a reference? Also as a comment on Figure 3: very convincing results! Well done.

We thank reviewer 2 for the compliment. We retrieved the STAT binding site motif from Mitchell et. al 2005 (DOI: [10.1111/j.1365-2567.2005.02091.x](https://pubmed.ncbi.nlm.nih.gov/15720432/)). We have now edited the main text (Line 181) and the method section (Line 389-391) to explain how we obtained the predicted binding motif and to include the reference.

Some suggested references that may be relevant to discussion in this study:

Sowa-Jasiłek et al:
 <https://www.sciencedirect.com/science/article/pii/S0944501316300799?via%3Dihub>
- A dedicated investigation of G. mellonella lysozyme activity against C. albicans

Marra et al:
<https://journals.asm.org/doi/full/10.1128/mbio.00824-21?rfr_dat=cr_pub++0pubmed&url_ver=Z39.88-2003&rfr_id=ori%3Arid%3Acrossref.org>
- A genetic deletion study in Drosophila that compares the effects of Lysozymes or AMPs on microbiome structure, finding AMPs control G- bacteria while Lysozymes control G+ bacteria

A couple additional references the authors could consider. Do the authors’ results provide insight into what is going on in these shrimp infection systems:
- Shrimp PGN:
 <https://journals.plos.org/plospathogens/article?id=10.1371/journal.ppat.1010967>
- anti-LPS factorD -> antiviral?:
 <https://link.springer.com/article/10.1007/s42995-021-00113-y>

We thank reviewer 2 for kindly suggesting these research articles.
